# Dermatomyositis: A Presenting Clinical Vignette in a Patient With Breast Cancer

**DOI:** 10.7759/cureus.10624

**Published:** 2020-09-23

**Authors:** Ali Allouch, Teisir B Zaatarikahale, Mohamad K Moussa, Youssef Jounblat, Nizar Bitar

**Affiliations:** 1 Oncology, Lebanese University, Faculty of Medical Sciences, Beirut, LBN; 2 Internal Medicine, Lebanese University, Faculty of Medical Sciences, Beirut, LBN; 3 Orthopedic Surgery, Lebanese University, Faculty of Medical Sciences, Beirut, LBN; 4 Hematology and Medical Oncology, Hopitaux Civils de Colmar, Colmar, FRA; 5 Hematology and Medical Oncology, Sahel General Hospital/Lebanese University Faculty of Medicine, Beirut, LBN

**Keywords:** dermatomyositis, amyopathic dermatomyositis, metaplastic breast cancer, paraneoplastic syndromes

## Abstract

Dermatomyositis (DM) is a rare idiopathic inflammatory myopathy, which is associated with malignancy in 15%-30% of cases. Breast cancer, the most frequent malignancy diagnosed in women, can feature uncommon presentations, such as paraneoplastic syndrome including DM. The aim of this case is to promote awareness regarding any adult patient who presents with DM for early detection and treatment of a possible underlying malignancy. Our patient was diagnosed and treated for DM without any improvement, until she presented to our department, and after a comprehensive history and physical exam, an underlying breast cancer was detected. It was metastatic unfortunately, but she improved after treatment with regression of symptoms related to DM.

## Introduction

Dermatomyositis (DM) is an idiopathic inflammatory myopathy characterized by proximal muscle weakness, rash, and other systemic manifestations. There is a well-established association between DM and malignancy, with 15%-30% of DM patients having an underlying malignancy [1-3].

The reported incidence for DM varies from 0.5 to 0.89 per 100,000/year [3], affecting mostly middle-aged women, at a 2:1 ratio with men in the same age group. Malignancies associated with myopathies have been reported in the medical literature since 1916 [4,5]. If present, the malignancy can precede, occur concurrently with, or follow the diagnosis of DM [2]. Both DM and polymyositis are associated with increased risk of developing malignancies. The diagnosis of DM is associated with different types of malignancies and differs between gender and countries. In Asia, mainly in southern China and Southeast Asia, nasopharyngeal cancer is the most common malignancy detected in male whereas breast cancer is the main malignancy in female. On the other hand, western countries have different epidemiology concerning DM, in which ovarian and colorectal and lung cancer were the two most common malignancies detected in patients with DM [6,7].

Herein, we present a patient diagnosed with DM, and subsequently diagnosed with metastatic breast cancer. Symptoms related to DM did not resolve until treatment for the underlying malignancy began. This case serves as a reminder that a common disease (breast cancer) may present with uncommon features (DM as a paraneoplastic syndrome), and physicians must consider malignancy as the underlying systemic process when an adult patient presents with DM.

## Case presentation

A 45-year-old female patient from Iraq, known to have a history of arthritis, presented for severe proximal muscle weakness, diffuse rash, and periorbital heliotropic rash. The patient was completely bedridden; she was only able to move her wrist and feet.

History extends back to three months prior to presentation, when the patient was diagnosed with DM based on clinical features and elevated creatine kinase, noting that the patient was started on prednisone and azathioprine without any improvement. The patient had refused muscle biopsy at the time of initial presentation. 

**Figure 1:**
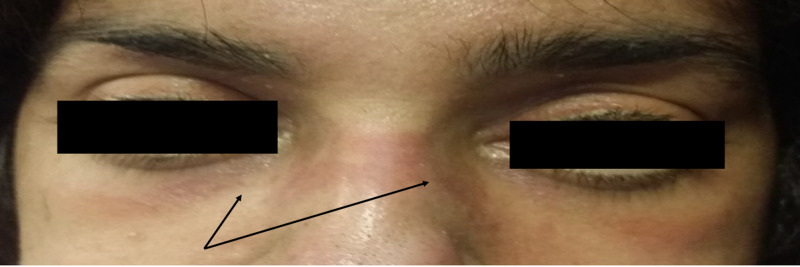
Photograph of the patient's face showing heliotropic rash and periorbital edema.

The patient reported dysphagia initially to solids but progressed to involve liquids also. Upon physical exam, she has diffuse rash and periorbital heliotropic rash (Figure 1). We palpated a firm mass in the right axillary region. The patient had severe proximal muscle weakness.

**Figure 2:**
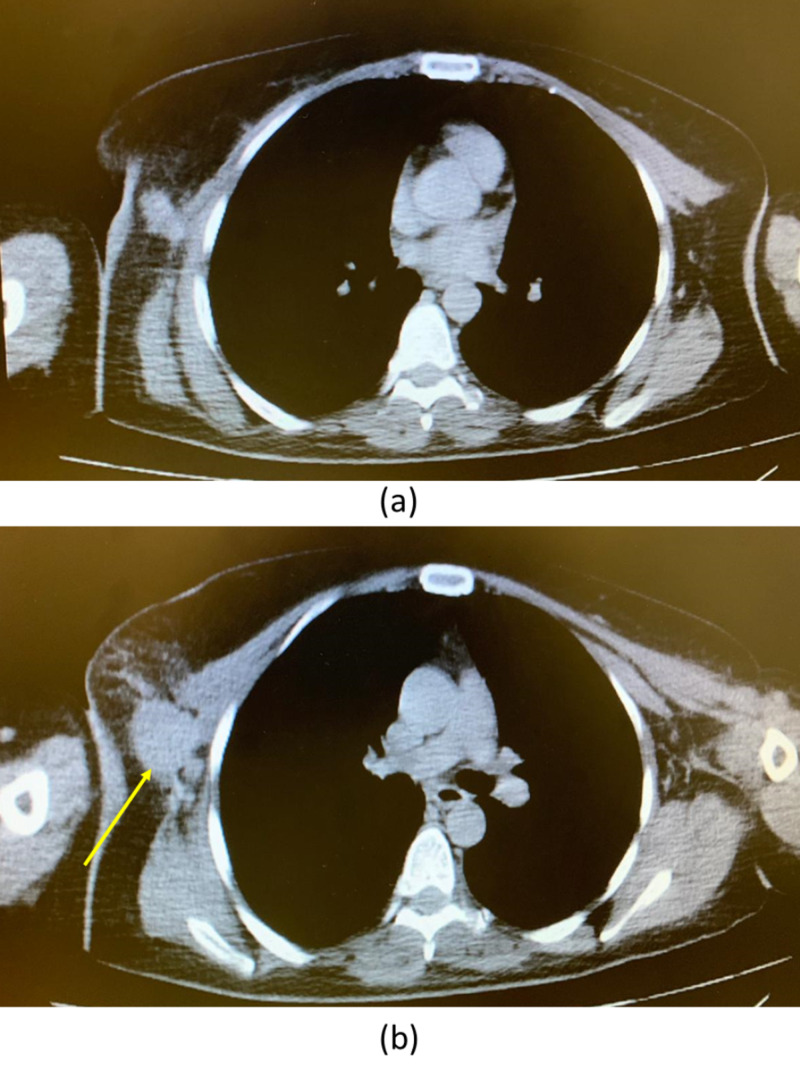
CT scan of the chest showed a 2.8-cm soft-tissue mass (yellow arrow) at the axillary right breast tail. (a) Axial cut above the lesion, and (b) axial cut through the lesion (yellow arrow).

CT scan of the chest, abdomen, and pelvis showed a 2.8-cm soft-tissue mass at the axillary right breast tail (Figure 2).

**Figure 3:**
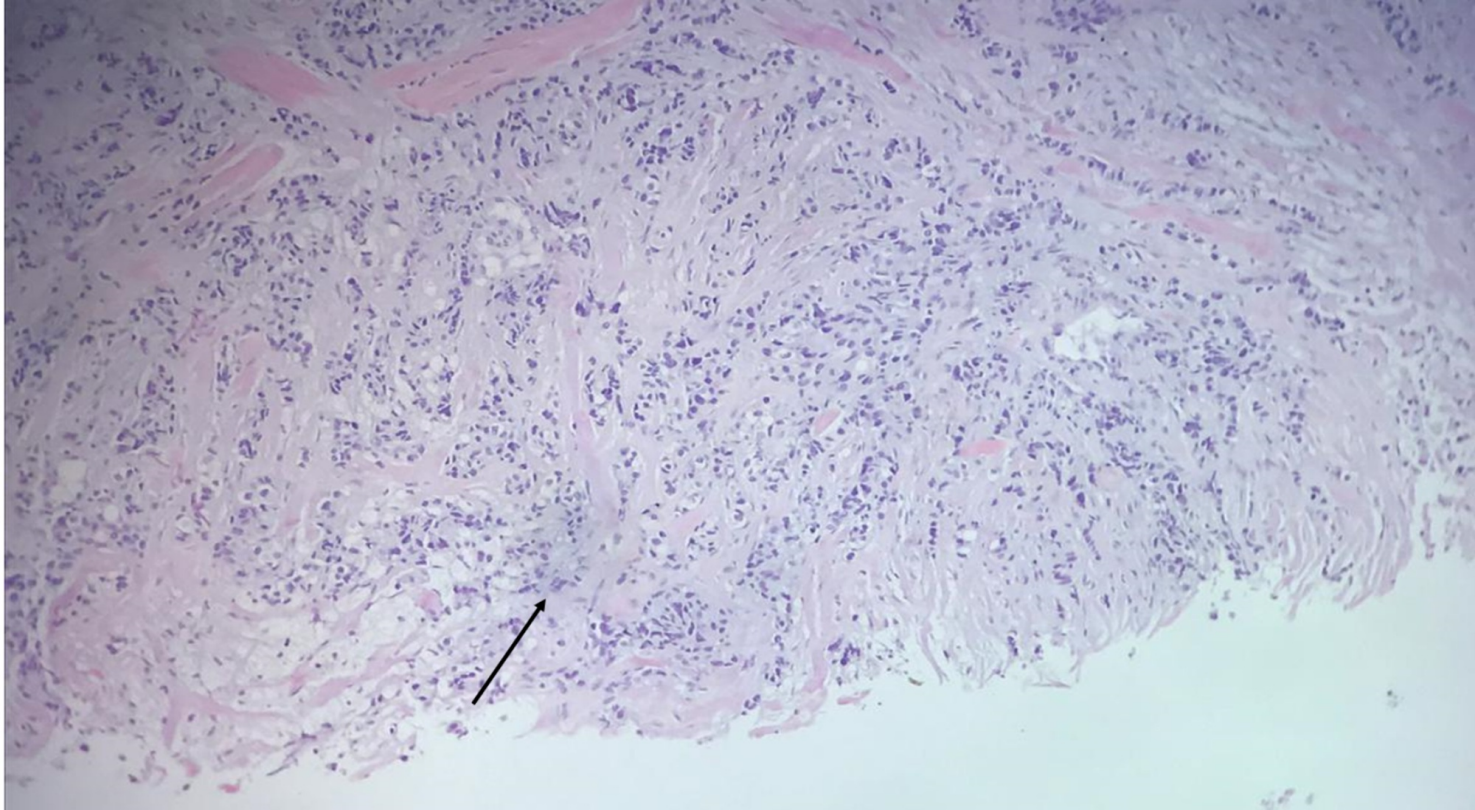
Pathologic examination with haemotoxylin and eosin stain shows neoplastic proliferation consisting of large cell with big nuclei arranged in tubes and trabeculae with moderate nuclear atypia and mitosis (black arrow).

Echo-guided biopsy of the axillary mass revealed a neoplastic proliferation consisting of large cell with big nuclei arranged in tubes, and trabeculae with moderate nuclear atypia and mitosis. The stroma is fibrotic and moderately inflamed, and no lymph node parenchyma is seen (Figure 3).

Immunohistochemical study revealed the following: positive for the gross cystic disease fluid protein (GCDFP) with focal positivity for cytokeratin 7 and mammaglobin. Immunohistochemical study for hormonal receptors: estrogen receptors are strongly positive (80%-85%), progesterone receptors are strongly positive (90%-95%), HER2 is positive (2+), fluorescence in situ hybridization (FISH) for HER2 negative, Ki67 labels 30% of nuclei. The overall pattern was consistent with invasive ductal carcinoma, grade II of Scarff-Bloom-Richardson, hormonal receptor positive, and HER2 negative.

Positron emission tomography (PET) scan showed two 18F-fluorodeoxyglucose (FDG) lesions at the upper outer quadrant of the right breast corresponding to soft tissue lesions, the largest measuring 2 cm associated with a maximum standardized uptake value (SUVmax) of 13.38, compatible with focal right breast malignant disease.

Moreover, several FDG-avid right axillary and subpectoral lymph nodes were identified, the largest measuring 3.1 cm with an SUVmax of 16.8, consistent with metastatic lymph nodes.

**Figure 4:**
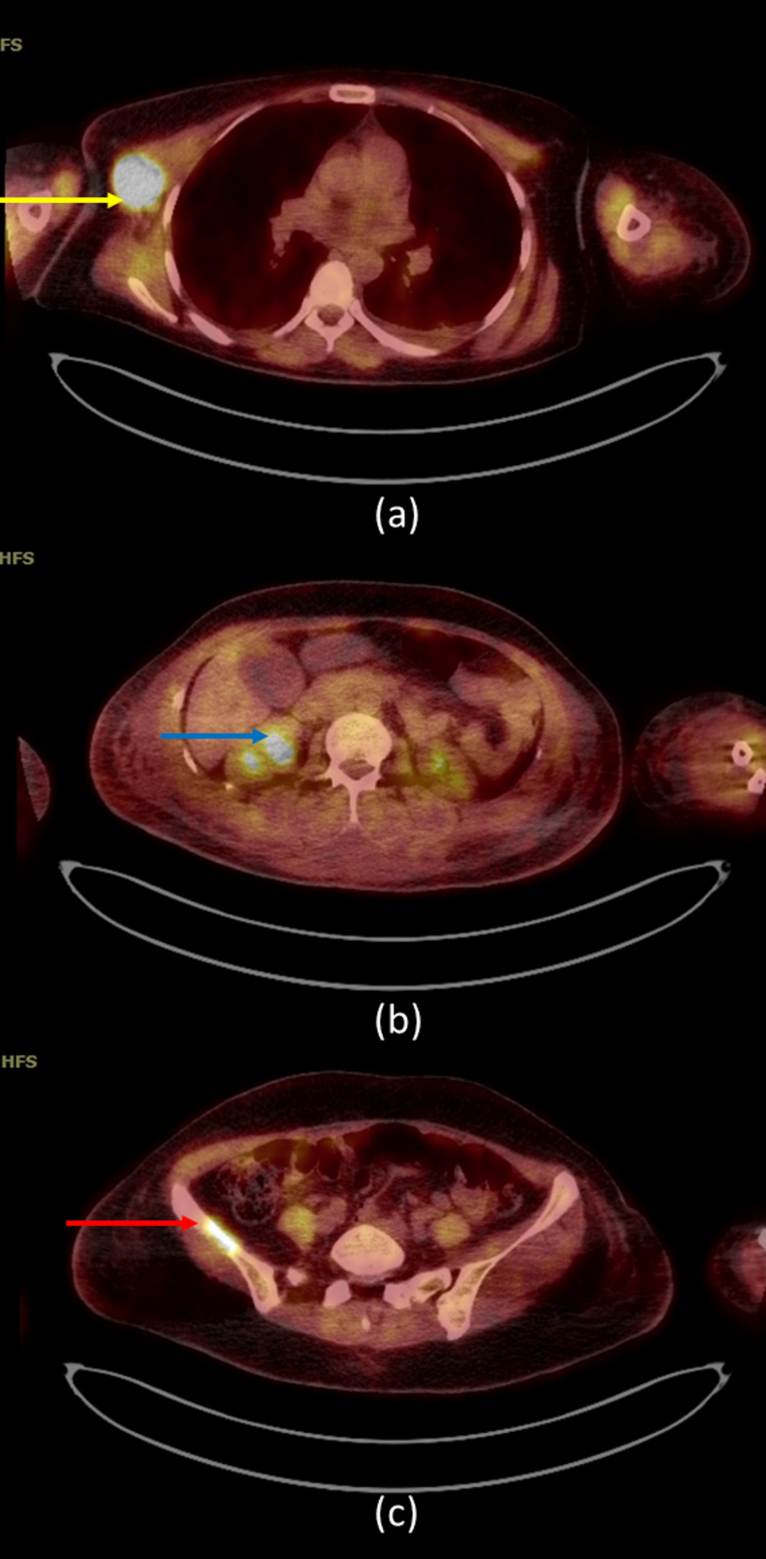
Positron emission tomography (PET) scan shows (a) two 18F-fluorodeoxyglucose (FDG) lesions at the upper outer quadrant of the right breast corresponding to soft tissue lesions (yellow arrow), with a maximum standardized uptake value (SUV)max of 13.38, compatible with focal right breast malignant disease; (b) FDG-avid right suprarenal lesion (blue arrow) with an SUVmax of 12.98, corresponding to right adrenal nodule, metastatic in nature; and (c) a large FDG-avid lytic lesion at the right iliac bone (red arrow) with an SUVmax of 7.92, also metastatic in nature.

There was an FDG-avid right suprarenal lesion with an SUVmax of 12.98, corresponding to right adrenal nodule, metastatic in nature with a large FDG-avid lytic lesion at the right iliac bone with an SUVmax of 7.92, also metastatic in nature (Figure 4).

This case was consistent with metastatic breast cancer (stage 4), hormonal receptor positive. The patient started chemotherapy for metastatic breast cancer.

A few days after the first cycles of chemotherapy, the patient clinical condition started improving, with a rapid regression of symptoms with regard to psychological condition, extremities motor function, head movement, dysphagia, heliotropic rash, periorbital edema, and upper and lower limb edema.

After a total of six cycles of chemotherapy received, the patient regained her motor functions; she was able to sit and stand alone as well as walk with assistance, albeit with some residual deficit in motor function. Then, she was started on hormonal treatment with medical ovarian suppression function, with no signs or symptoms of recurrence 18 months after starting treatment.

## Discussion

Globally, breast cancer is the second-most frequently diagnosed malignancy just behind lung cancer, accounting for over two million cases each year [8]. It is the primary cause of cancer-related deaths in women worldwide. Breast cancer usually presents with a palpable mass, abnormal mammogram during screening time, or symptoms related to metastases. It rarely manifests as a paraneoplastic syndrome, like DM [9].

Paraneoplastic syndromes encompass a wide range of disorders due to an altered immune system from an established malignancy. Underlying cancer cells are thought to produce autoantibodies, hormonal signals, or cytokines that affect a vast array of organ systems. Signs and symptoms of these disorders are chronologically dissociated from the date of the cancer diagnosis. DM is a well-known paraneoplastic syndrome that affects muscular and dermatological systems. Out of all DM cases studied by Goyal and and Nousari, 15%-30% were paraneoplastic [10].

DM is a rare idiopathic inﬂammatory myopathy demonstrated by multiple skin ﬁndings, proximal muscle weakness, and pain. It is usually associated with activation of autoreactive T lymphocytes, downregulation of T regulator cells, and release of proinflammatory cytokines leading to B and T cells tolerance loss [11]. The molecular mechanisms underlying these associations are still unknown. Some specialists hypothesize that a possible antigenic similarity between regenerating myoblasts and some cancer cell population might trigger the inflammatory response [11-13].

To our knowledge, there is no lab test to differentiate between the idiopathic and the malignant associated DM. It is a rare diagnosis, and there is little evidence to guide treatment until now [14]. No prospective data on how to manage those patients are comprehensively available; in general, however, we should follow the standard guidelines for treatment of metastatic breast cancer [9].

There is no clear consensus on the role of preoperative chemotherapy or hormonal therapy in patients diagnosed with early or locally advanced breast cancer and DM. Diseased skin (from DM inflammation or infection) represents a major challenge postoperatively, especially when dealing with wound healing. In such cases, if possible, skin condition should be treated and ameliorated before surgery. Otherwise, if the skin is not diseased, physician can proceed with neoadjuvant treatment before surgery [5,15,16]. Also concerning metastatic breast cancer, there is no data concerning role of surgery in patient with DM and breast cancer.

A total of 44 previously published cases of DM in patients with breast cancer were identified; however, only 22 patients had specific staging and a confirmed DM diagnosis.

Luu et al. reported a patient with DM and breast cancer, highlighting the rapid progression and regression of symptoms related to DM after treatment of breast cancer, thus emphasizing the benefit of early diagnosis and treatment of DM as well as the underlying breast cancer [17].

Sandhu et al. also reported a patient diagnosed with DM and breast cancer. The treatment of breast cancer led to the regression of muscle weakness, rash, and other symptoms related to DM [18].

Osako et al. reported a specific clinical scenario: the patient was diagnosed and treated for DM and breast cancer. The treatment was followed by a regression of the symptoms. A few months later, the patient had a flare-up of skin manifestations with elevated creatine kinase. Immediate workup showed recurrence of breast cancer [19].

## Conclusions

DM is a rare autoimmune disease, with unknown etiology. There is a well-established association between DM and malignancy in 30% of cases. All patients newly diagnosed with DM should be evaluated for the possibility of an underlying malignancy. A comprehensive history and physical examination, including a pelvic exam, along with the appropriate investigations should be done. Furthermore, the specific association between breast cancer and DM has not been well characterized. No guidelines for the management of DM in breast cancer have been established. In most cases reported in the literature, the treatment of breast cancer leads to the regression of symptoms related to DM, and the recurrence of symptoms may be a sign of recurrence of the underlying malignancy, reinforcing the value of additional workup for early detection and treatment.
